# The leaf‐footed cactus bug is not a cactus specialist: *Narnia femorata* feeds, fights, and mates on thistle

**DOI:** 10.1002/ece3.70257

**Published:** 2024-10-08

**Authors:** Lauren A. Cirino, Isaac McEvoy, Logan C. Smith, Zachary Emberts

**Affiliations:** ^1^ Department of Integrative Biology Oklahoma State University Stillwater Oklahoma USA; ^2^ Department of Biological Sciences University of Mary Washington Fredericksburg Virginia USA

**Keywords:** Coreidae, host range expansion, host shift, insects, sexual selection

## Abstract

Novel host plants are incorporated into the diets of phytophagous insects when females oviposit and juveniles feed and survive on them. A change in diet, however, can have morphological consequences. We recently found a population of the leaf‐footed cactus bug, *Narnia femorata* (Hemiptera: Coreidae), a historical cactus specialist, living and feeding on *Cirsium* thistle. We also found adults breeding and males using their enlarged hind legs (i.e., weapons) in male–male combat on thistle. When we compared this thistle population with a population feeding on cactus, we found that both populations had similar body and weapon sizes as well as weapon composition. However, the population living on thistle had longer mouthparts than the population found on cactus, although this difference only occurred at larger body sizes. This difference in adult mouthpart size is likely a result of the juvenile rearing environment (i.e., thistle or cactus). However, genetic differences may also affect this trait. Our results provide some interesting avenues for future research (e.g., a reciprocal transplant experiment) in a species with a recent host range expansion.

## Graphical abstract

We recently found a population of the leaf‐footed cactus bug, *Narnia femorata* (Hemiptera: Coreidae), a historical cactus specialist, living on thistle. When we compared this thistle population with a population feeding on cactus, the population living on thistle had longer mouthparts than the population found on cactus, although this difference only occurred at larger body sizes. This difference in adult mouthpart size is likely a result of the juvenile rearing environment (i.e., thistle or cactus) and suggests a host plant range expansion for the species.

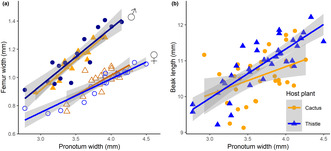

## 1 INTRODUCTION

Many phytophagous insects are host–plant specialists (Bernays & Chapman, 1994). If insects are to expand or shift their host plant diet, they must be able to recognize a new host plant as food, survive, and reproduce when feeding on it (Rausher, 1983). Host range expansions can occur through bottom‐up forces due to host plant availability, detectability, and suitability for the insect species (Miller, 2008; Vidal & Murphy, 2018). Alternatively, predation and parasitism may force insects to shift to other host plants to escape these top‐down forces (Bernays, 1988; Bernays & Graham, 1988; Price et al., 1980; Vidal & Murphy, 2018). No matter the reason, switching host plants can result in behavioral and/or morphological changes to the insects (Ashra & Nair, 2022; Bowers et al., 1992; Zovi et al., 2008). These host plant shifts or expansions are important to investigate because they may contribute to our understanding of the evolution of insect–plant relationships and speciation of phytophagous insects.

*Narnia femorata* has historically been categorized as a cactus plant specialist (Cirino & Miller, 2017; Vessels et al., 2013). Populations feed on several cactus plant species such as *Opuntia*, *Cylindropuntia*, *Ferocactus*, and *Cereus* (Vessels et al., 2013). Their range roughly mirrors the range of cactus plants from the southern United States to central America (Baranowski & Slater, 1986; Brailovsky, 1975; Brailovsky et al., 1994; Mann, 1969; Palomares‐Perez et al., 2012). *N. femorata* juveniles and adults feed mostly on the fruit of cactus plants and are found in abundance on cactus plants with ample fruit (Miller, 2008; Miller et al., 2006), although they also feed on the cactus pads (Cirino, 2023; Nageon‐De Lestang & Miller, 2009). *Opuntia* cactus pads differ in nutritional composition from cactus fruit (Barbera et al., 1992; Rodriguez‐Felix & Cantwell, 1988). Cactus pads generally represent a poor diet for *N. femorata* compared to cactus pads with fruit (Cirino, 2023; Nageon‐De Lestang & Miller, 2009). Thus, a single cactus plant can vary in nutrition across the season as it develops fruits (Barbera et al., 1992; Cirino & Miller, 2017).

**FIGURE 1:**
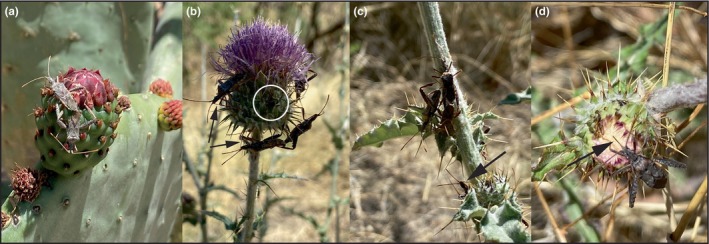
(a) *Narnia femorata* in copula on the flower bud of a cactus plant. (b) Four adults (one mating pair) and one juvenile (in white circle) on the flower of a thistle plant. Two adults are feeding on the thistle flower. (c) An aggregate of adults with one mating pair on a thistle plant stem. One adult is feeding on the stem of the thistle. (d) An adult *N. femorata* feeding on a thistle flower (all arrows point at the beaks of the insects feeding). Photos taken five days prior to collection of the *N. femorata* thistle population (b–d). Photo credit: Zachary Emberts.

When *N. femorata* feeds on a single host plant species, there can be drastic differences in adult behavior and morphology due to the plant's phenology (Cirino et al., 2022; Cirino & Miller, 2017; Woodman et al., 2021). *N. femorata* males engage in male–male competition and use their hind legs with enlarged and spiny femurs as weapons to win territory and eventually mate (Procter et al., 2012). The quality of the territory in which they fight over influences the likelihood that males will fight (Nolen et al., 2017) and both the territory quality and the nutrients that *N. femorata* ingests affects mating partner decisions (Figure 1a, Addesso et al., 2014; Cirino, 2023; Gillespie et al., 2014; Wilner et al., 2020). Poor diets decrease juvenile survivorship and prolong development (Cirino, 2023; Cirino et al., 2022; Nageon‐De Lestang & Miller, 2009). If *N. femorata* survives to adulthood on these poor diets, they grow into adults that have smaller bodies (Cirino, 2023; Miller et al., 2016; Sasson et al., 2016), testes (Joseph et al., 2016; Sasson et al., 2016), ovaries (Cirino, 2023), produce fewer eggs (Cirino, 2023; Cirino et al., 2022), and have smaller hind femur weapons (Miller et al., 2016; Sasson et al., 2016; Woodman et al., 2021) that have weaker structural integrity (Woodman et al., 2021) compared to those insects raised on a good diet. An improved diet, however, can help partially rescue some reproductive traits (i.e., ovary size and egg number) compared to those that had a lifetime of a poor diet (Cirino, 2023; Cirino et al., 2022; Wilner et al., 2020).

Some studies have also quantified morphological differences when *N. femorata* feed on different cactus species (Allen et al., 2021; Allen & Miller, 2017). When *N. femorata* feeds on a novel cactus host (*Opuntia robusta*) rather than their local cactus host (*Opuntia mesacantha* spp. *lata*), sexual dimorphism increases for body size and decreases for hind leg weapons because males become tiny adults with female‐like hind legs (Allen & Miller, 2017). Moreover, individuals feeding on *O. engelmannii var. lindheimer* grew longer mouthparts (i.e., beaks) presumably to penetrate the thicker walls of the fruit and access the sugary rich nutrition at the center (Allen et al., 2021).

Here, we discovered *Narnia femorata* living on *Cirsium* thistle, a novel host plant for this species. We define host plants for this study as plants where animals live, feed, and breed. Thus, these host plants support the growth and development of juveniles and provide food for adults. Cactus and thistle plants are distantly related to one another as the clades that they belong to (Caryophyllales and Asterales, respectively) are estimated to have diverged from one another approximately 115 Mya (Zeng et al., 2017). We hypothesized that this is a host plant shift by a population of *Narnia femorata* located in Arizona, USA, and an overall host range expansion by the species. This hypothesis predicts that juveniles and adults will be found on thistle and not on cactus in some areas while in other areas found on cactus plants. Furthermore, it predicts that juveniles and adults will be feeding on the novel host plant and adults will be mating and fighting on it. Finally, we predict there will be at least some morphological differences between populations living on thistle compared to cactus similar to those found in previous novel cactus host studies.

In this study, we found and noted the life stages and behaviors of *N. femorata* on thistle and cactus plants at two field sites in Arizona, USA (Figure 1). We also measured important morphological traits related to fitness. Finally, we examined photographs of *Narnia femorata* in the iNaturalist database and identified the substrate they were living on and if they were feeding on a non‐cactus plant to determine if potential host plant changes have been documented previous to our survey. Our goal here was to estimate how long *N. femorata* has been feeding on thistle.

## 2 METHODS

We searched for *Narnia* at two field sites in Arizona, USA: the University of Arizona (UA) main campus (32.229301, −110.954619) and the Santa Rita Experimental Range (31.792675, −110.881673). The Santa Rita Experimental Range is about 41 miles (ca. 66 km) south of the University of Arizona main campus. We surveyed the wide variety of cacti present on the UA main campus including *Carnegiea gigantea*, *Opuntia* cf. *ficus‐indica*, *Ferocactus wislizeni*, *Pachycereus marginatus*, and *Cleistocactus* cf. *hyalacanthus*. No thistle was present at the UA site. At the Santa Rita Experimental Range site, we surveyed the two species of cacti present within the vicinity (*Opuntia* cf. *ficus‐indica* or *Ferocactus wislizeni*) as well as *Cirsium* cf. *neomexicanum* thistle. Two people surveyed the field sites for 1 h and noted the life stages and behaviors of *N. femorata*. We surveyed the UA field site multiple times and collected hundreds of *N. femorata* from cactus plants from March through April of 2022 for use in another experiment. Of these, we randomly chose 36 (18 of each sex) to compare *N. femorata* morphology from the population we found on thistle at the Santa Rita Experimental Range (*n* = 36) which we collected in May of 2022. We used the *Narnia* dichotomous key and confirmed the insects found at both sites were *N. femorata* (Brailovsky & Barrera, 2013).

We cold euthanized the insects we collected and stored them in a freezer at −18°C until we photographed them. We removed the hind legs of each insect by carefully gripping the proximal position on the leg and twisting it at the trochanter‐femur joint to prevent any femur damage while still removing the leg from the body. We positioned each insect's body, dorsal side up, and detached hind leg atop a light box next to a scale bar and photographed them using a digital camera (Canon EOS 7D, Canon, Tokyo, Japan). This positioning allowed us to capture the pronotum and hind femur of each insect. We also took a ventral side up photograph of their bodies to capture their mouthparts (i.e., beaks). We then measured pronotum width, right hind femur width, and beak length using ImageJ (version 1.53t; Abràmoff et al., 2004). We measured pronotum width because it is a good proxy for body size in this species (Gillespie et al., 2014). We measured hind femur width because it is the portion of the leg that is enlarged with spines and used as a weapon in male–male competition (Miller et al., 2016; Procter et al., 2012). Furthermore, all traits that we measured in this study can vary in size based on their host plant diet (Allen et al., 2021; Cirino, 2023; Cirino & Miller, 2017; Miller et al., 2016).

We then measured puncture resistance of the hind femur using a penetrometer (December 12, 2023–January 18, 2024). The penetrometer simulates a spine from a rival male's leg pressing and squeezing until it punctures the leg of its opponent (Woodman et al., 2021). Hind legs are used in male–male competition in this species to acquire territory and access mates (Procter et al., 2012). If a leg is injured (e.g., punctured) during combat, males may need to drop the leg (i.e., autotomize) or they are likely to suffer mortality (Emberts et al., 2017). If they autotomize an injured hind leg, they are more likely to become subordinate when they compete for territory and have fewer opportunities to mate (Emberts et al., 2018). Thus, structural integrity of the hind leg is likely crucial to their reproductive success. We removed approximately a 1 × 2 mm section of the hind leg's cuticle. We placed the leg samples, one at a time, in a sample holder of an Imada penetrometer that had a central circular hole diameter of 1.2 mm. We then put the sample holder under a ZTA‐1 programmable digital gauge (accuracy of 11 mN) to quantify the force it takes to puncture this body part (i.e., structural integrity). We used a size three Austerlitz insect pin and set the force gauge to move at a constant speed of 35 mm/min (similar to Woodman et al., 2021) using an Imada EMX‐275 (USA) vertical motorized test stand. We used Imada's Force Recorder Data Acquisition Software to obtain puncture resistance measurements.

To ensure we did not overlook the *N. femorata* host plant range expansion in the last several years, we examined the extent to which *N. femorata* were publicly identified to be living and feeding on cactus and other plants using the iNaturalist database (https://www.inaturalist.org/). iNaturalist is a crowdsourced biodiversity information system that is hosted by an independent and non‐profit organization (iNaturalist, 2023). Community users can upload observations (e.g., photos) using the application on a smartphone or the website portal. Photos are then geotagged and assigned the observation date. The automated species identification algorithm helps users identify the organism, which utilizes available taxa information from the iNaturalist database (Van Horn et al., 2018). Once the observation is live, other iNaturalist community members can validate the identification and/or help identify the organism down to the lowest possible taxonomic clade. This crowdsourced method of identification leads to a >90% accuracy of species identification (e.g., Garretson et al., 2023).

We exported all *Narnia* observations from the iNaturalist website on November 28, 2023. We removed all observations (*n* = 1966) that were not categorized as *Narnia femorata*. We then examined each observation and identified the substrate that it was photographed on (any cactus plant, another plant type, or non‐living substrate). We removed the few observations that were obvious duplicates (*n* = 2), and we removed observations where we were unable to determine the substrate (*n* = 5). If the insect was photographed on another plant type, we recorded whether the mouthparts were in the plant.

### 2.1 Statistical analysis

To explore whether the host plant that *N. femorata* was found on influenced fitness‐related morphological traits, we constructed four linear models each with a gaussian distribution. For the first model, we included pronotum width (PW) as the dependent variable and host plant (thistle and cactus plants) and sex (female and male) as the independent variables. For the second and third models, we used beak length and hind femur width (FW) as the respective dependent variables. Our independent variables for these two models were host plant, sex, and pronotum width. In our final model, we included hind femur puncture resistance as the dependent variable and host plant, sex, and hind femur width as the independent variables. We included hind femur width instead of pronotum width in this model because puncture resistance (i.e., insect cuticle thickness) is expected to increase with the size of the structure (e.g., Peeters et al., 2017). We also initially included all 2‐way interactions in our models. However, we removed interactions when *p* > .15 in a stepwise fashion (Gotelli & Ellison, 2013). All analyses were performed using the *car* package v. 3.1‐2 (Fox & Weisberg, 2023) in R v. 4.3.2 (R Core Development Team, 2023).

## 3 RESULTS

We found *Narnia femorata* late‐stage juveniles and adults feeding on *Cirsium* cf. *neomexicanum* thistle at the Santa Rita Experimental Range (Figure 1b). Adults were also observed mating and fighting on the thistle at this site (Figure 1b–d and Video 1). Interestingly, *N. femorata* at this site were not observed on the nearby *Opuntia* cf. *ficus‐indica* or *Ferocactus wislizeni* cacti we surveyed. However, at the University of Arizona main campus, we found *N. femorata* juveniles and adults feeding on cacti. The vast majority were found on *Carnegiea gigantea*, but some individuals were also found on *Opuntia* cf. *ficus‐indica*, *Ferocactus wislizeni*, *Pachycereus marginatus*, and *Cleistocactus* cf. *hyalacanthus* cacti. Adults were observed mating and fighting on cacti at this site too (Figure 1a).

**VIDEO 1:** An aggregation of adults living, feeding, mating, and fighting on thistle. Two males fight on top of the currently copulating pair on the stem of the thistle (center frame of the video). The first fight starts at 33 s and the second fight starts at 57 s. The winning male tries to dislodge the current mating male from the female at 1 min and 44 s.

**FIGURE 2:**
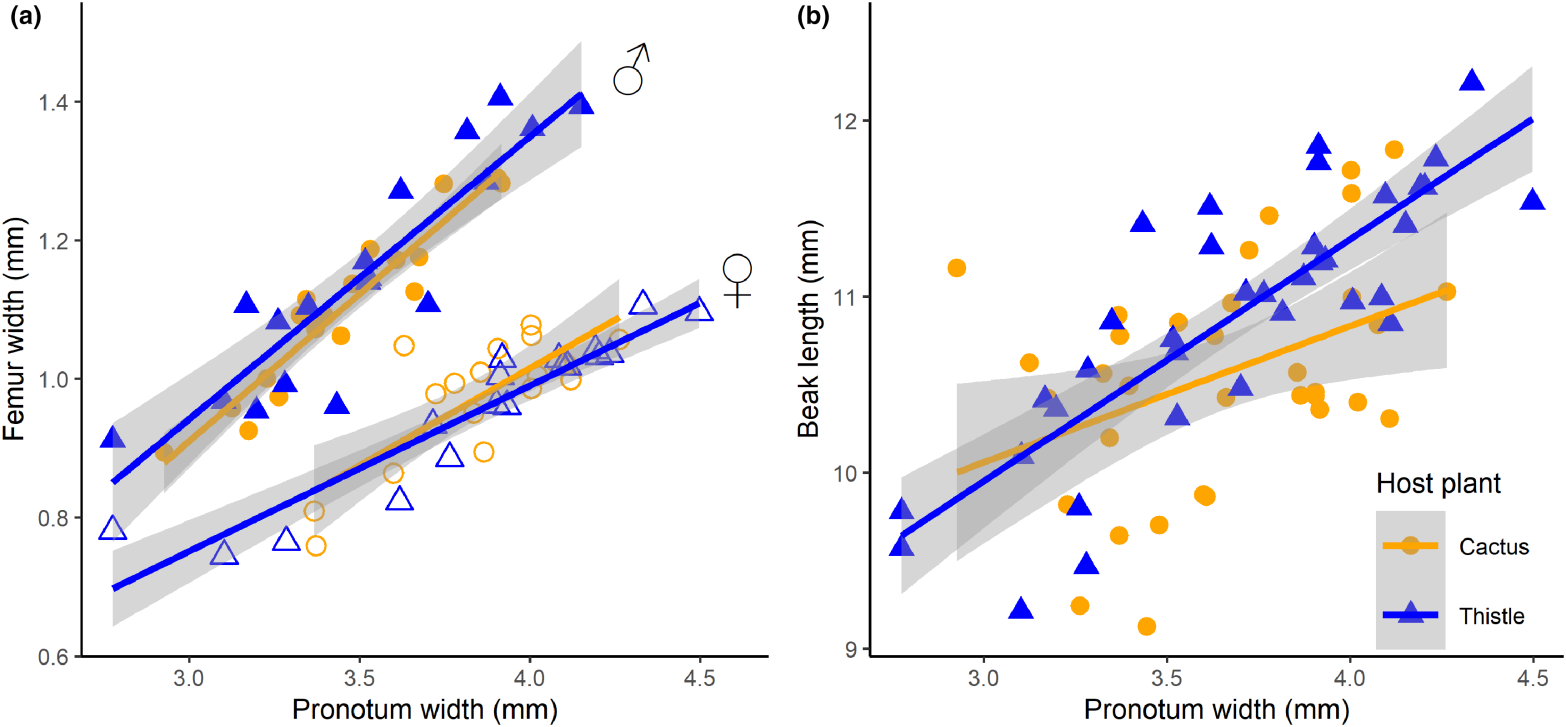
(a) Femur width increases with body size and this trait is sexually dimorphic in *Narnia femorata*, but it does not change based on the host plant that *N. femorata* was found on. (b) Large *N. femorata* found on thistle have longer beaks than large insects found on cactus. Shaded regions represent 95% confidence intervals.

**FIGURE 3:**
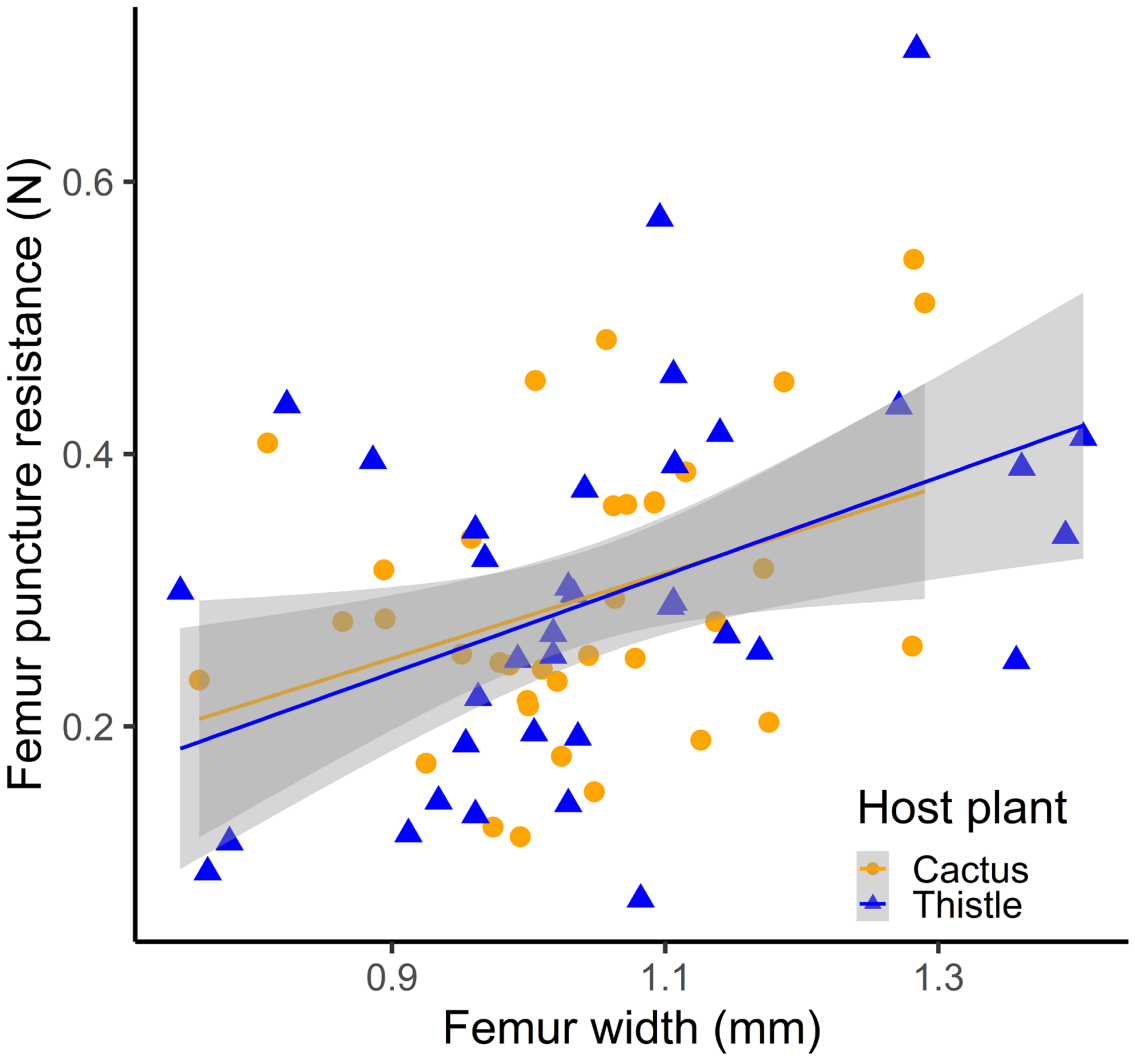
Femur puncture resistance (i.e., structural integrity) increases as femur width increases and was not affected by sex or by the host plant that *Narnia femorata* was found on. Shaded regions represent 95% confidence intervals.

When we examined morphological traits between the insects we found on cactus and thistle, we found a difference in beak length. However, this difference was size dependent. Large individuals found on thistle had longer mouthparts (i.e., beaks) than large individuals found on cactus (Table 1, Figure 2b). Beak length was similar for smaller individuals, regardless of what host plant they were found on (Table 1, Figure 2b). Furthermore, we found no differences in body size, weapon size, nor structural integrity of the hind leg weapon based on host plant (Tables 1 and 2; Figures 2a and 3).

**TABLE 1:** Results from three linear models examining morphological traits of *Narnia femorata* populations feeding on cactus and thistle.

Source	Df	Pronotum width		Femur width		Beak length	
	1	*F*	*p*	*F*	*p*	*F*	*p*
Sex	1	**23.64**	**<.001**	**297.89**	**<.001**	**4.52**	**.037**
Host plant	1	0.19	.668	3.68	.059	**4.03**	**.049**
Pronotum width (PW)	1	–	–	**115.40**	**<.001**	2.66	.108
Host plant × PW	1	–	–	3.59	.062	**5.13**	**.027**

*Note*: Significant terms are bolded. Color shading delineates the three different statistical analyses conducted for the morphological traits that were measured.

**TABLE 2:** Results from a linear model examining hind femur puncture resistance of wild‐caught *Narnia femorata* found on cactus and thistle plants.

Source	df	Femur puncture resistance	
		*F*	*p*
Sex	1	0.54	.467
Host plant	1	0.04	.843
Femur width (FW)	1	**4.56**	**.036**
Host plant × FW	1	0.03	.860

Note: Significant terms are bolded.

**FIGURE 4:**
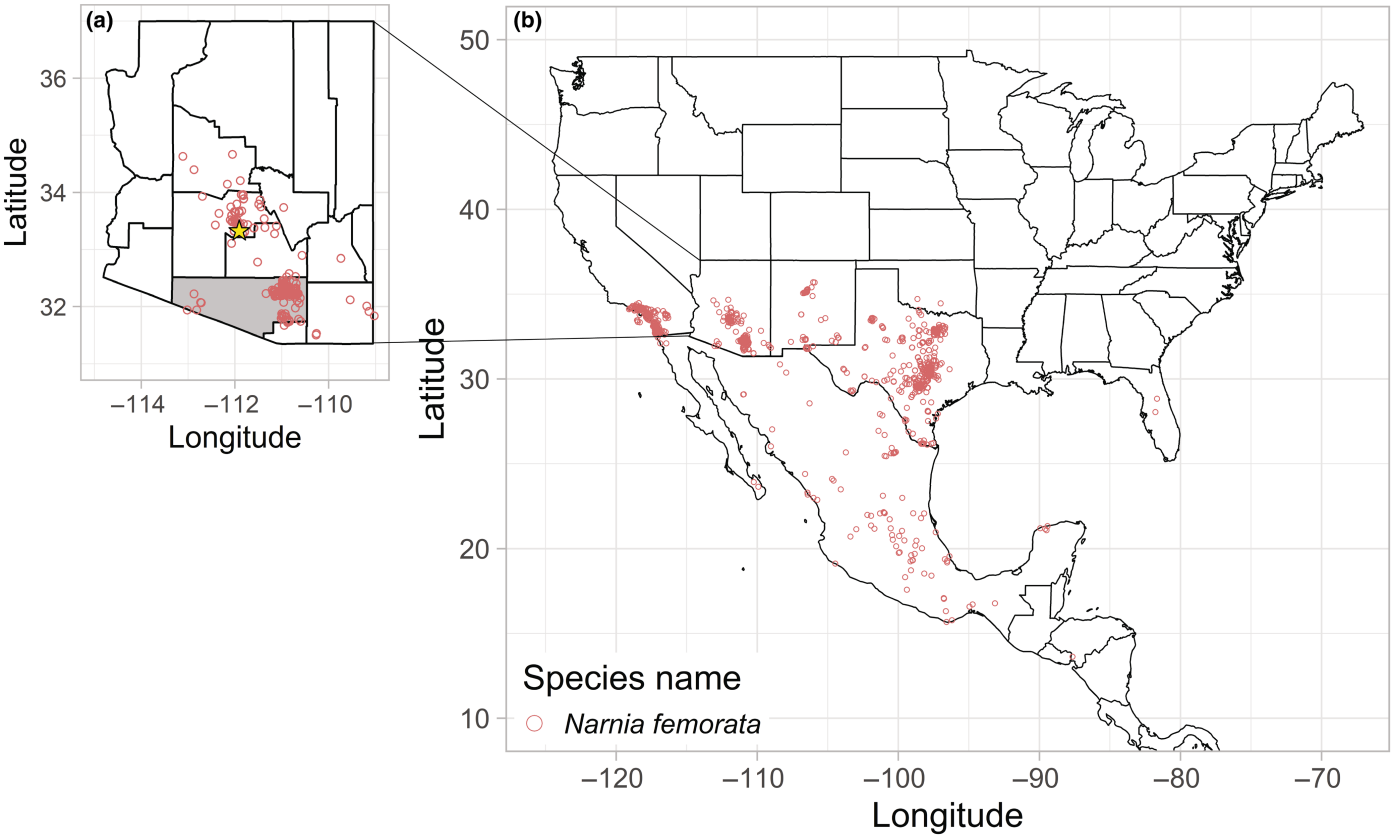
(a) Zoomed in map of Arizona with all counties delineated. Pima county, highlighted in gray, is where our two field sites for this study were located. The colored open circles represent the observations from iNaturalist of *Narnia femorata* that we pulled for this study. The one observation of *N. femorata* found feeding on a non‐cactus plant is indicated with the yellow star. (b) Map of the United States of America, Mexico, and central American countries where iNaturalist observations of *N. femorata* were recorded. All coordinates were pulled from observations made in the iNaturalist crowdsourced species identification system and mapped using R.

We found 1499 observations of what is identified as *Narnia femorata* on the iNaturalist website (Figure 4). Of the 1499 observations, 1334 were found on cactus plants (~89%) and 150 (~10%) were found on substrates that appeared non‐living (e.g., concrete, building). Fifteen (~1%) were found on non‐cactus plants. In all fifteen observations, the individuals were alone and only one insect appears to have its mouthpart inserted in the plant. This observation was added to iNaturalist on November 12, 2019, near Phoenix, Arizona, USA (Figure 4a).

## 4 DISCUSSION

We found *Narnia femorata* adults, a previously known cactus plant specialist, feeding, breeding, and fighting on *Cirsium* thistle. We also found late‐stage juveniles feeding on thistle. The consequences of switching to a novel food source may affect important fitness related traits. When we compared morphological traits between adults found on thistle and cactus, we found a difference in mouthpart (i.e., beak) length but no differences between any other morphological trait we measured. This difference suggests that *N. femorata* beaks were affected by their developmental diet so that they could reach the nutrients necessary to build their bodies. Our data supports our hypothesis that the population of *N. femorata* at the Santa Rita Experimental Range has made a host plant shift from cactus to thistle and suggests a host range expansion for the species. Future work should examine the survival, development, and reproduction of this species in a reciprocal transplant experiment to help us understand the extent to which population genetics and the host environment influence the morphology of these insects and whether a host range expansion has indeed occurred.

Novel host plants can be incorporated into phytophagous insects' diets if females can locate the plants and oviposit on them (Bowers et al., 1992; Rausher, 1983). Additionally, the juveniles that hatch on these novel plants must accept the plant as food and be able to grow and develop (Bowers et al., 1992; Rausher, 1983). We found evidence that *N. femorata* females can locate thistle plants since they were found feeding and mating on these plants. We also found late‐stage juveniles on thistle plants. Since juveniles of this species do not possess fully formed wings until the adult stage, their mobility is restricted to the place in which they hatched (Vessels et al., 2013). Thus, observing late‐stage juveniles on thistle plants suggests that females have oviposited on thistle, those eggs have hatched, and juveniles have adequately developed on these plants. We found juveniles and adult *N. femorata* living on cactus plants on the University of Arizona main campus, but we did not observe any living on cactus plants at the Santa Rita Experimental Range during our survey. Together, our observations indicate that the *N. femorata* population at the Santa Rita Experimental Range may have switched hosts but because *N. femorata* still lives and feeds on cactus plants in other locations, this suggests a species level host range expansion.

It is possible that the juvenile and adult *N. femorata* that we observed on thistle did not live their entire lives on this plant. Adult *N. femorata* possess wings and they can fly to other plants. *Cirsium neomexicanum* thistle is predominantly found throughout the southwestern United States and northern Mexico (Shreve & Wiggins, 1964), which overlaps with the range of many cactus species (Drezner, 2014; Novoa et al., 2015; Weniger, 1969) and *N. femorata* (Figure 4). Indeed, we found thistle and cactus plants growing near one another at the Santa Rita Experimental Range. The adults that we found on thistle may have lived their juvenile lives on cactus plants and then made a switch to thistle as adults or switched back and forth as nutritionally needed. Similarly, juveniles could have walked from the cactus patches where they hatched to the thistle plants we found them on, although this would be quite treacherous as they are heavily predated upon (Piovia‐Scott et al., 2017). Regardless, plant choice can change throughout an insect's life (Rausher, 1983), so changes to diet during the juvenile or adult stage could have occurred forcing these insects to switch host plants.

One of the barriers to incorporating a new host plant into an insect's diet is their capacity to successfully consume it (Bowers et al., 1992; Rausher, 1983). Thus, mouthpart morphology is crucial for food consumption. It is possible that the morphological differences we found in mouthpart length between populations are due to differences in developmental diet (i.e., thistle versus cactus plants), especially since no juvenile or adults were found on nearby cacti. Mouthpart plasticity is common when *N. femorata* switches diets (Allen et al., 2021; Cirino & Miller, 2017). When *N. femorata* switches from one species of cactus plant to another (Allen et al., 2021) or when *N. femorata* switches from early to late‐season cactus diets within the same cactus species, mouthpart length is affected (Cirino & Miller, 2017). Longer mouthparts presumably help *N. femorata* access the sugary rich center of the cactus fruits when feeding on a larger or thicker cactus fruit (Allen et al., 2021; Cirino & Miller, 2017). The need for longer mouthparts when insects feed on thistle might be necessary to access the nutrient rich middle of thistle reproductive heads. Once fertilized, the reproductive heads of *Cirsium* thistle can hold hundreds of small fruits (i.e., achenes – single seed fruits; e.g., *Cirsium vulgare*: Michaux, 1989). Since the fruits are small and numerous, they may be challenging to access with shorter mouthparts and thus growing longer mouthparts may be beneficial to accessing the nutrition within them. However, *N. femorata* was also observed feeding on the stem of *Cirsium* thistle as adults (Figure 1), and it is unclear what the nutritional importance of each of these plant parts is to *N. femorata* growth and development. Furthermore, the insects we collected were from two different field sites and the genetic differences between these populations could explain the difference we observed in mouthpart length (Allen et al., 2021). Future work should tease apart the effects of diet and genetic factors on mouthpart length in these populations.

We were surprised to find *N. femorata* living on thistle plants since *N. femorata* and its close relative, *Narnia snowi*, are historical cactus plant specialists (Fernandes et al., 2015; Mann, 1969) and have been documented living and feeding on cactus plants since 1912 (*N. femorata* and *N. pallidicornis* were combined retaining *femorata* as the species name: Gibson & Holdridge, 1918; Hunter et al., 1912). Current phylogenetic analyses suggest that *N. femorata* and *N. snowi* diverged more than 250,000 years ago (Miller et al., 2024). Our data provides evidence for a host range expansion for *N. femorata* on thistle as early as 2021. However, the iNaturalist observation that shows *N. femorata* feeding on another non‐cactus plant provides some (albeit weak) evidence that a host range expansion could have started as early as 2019. We note that although *N. femorata* was photographed with its mouthparts in a non‐cactus plant, it does not necessarily mean that they are getting all the necessary nutrients to sustain their lives from this plant (i.e., this may not be a host plant for this insect species as defined for this study). Since *N. femorata* and *N. snowi* are sister taxa, the *N. femorata* lineage may have been a cactus specialist for over 250,000 years and only recently expanded its host plant range. There is a small chance that the population of *N. femorata* that we found at the Santa Rita Experimental Range could be a new species that has yet to be described. However, we have personally observed unmated individuals from each population mate and produce viable offspring with members of the other populations. Even with this information, it is still important that future work examines this new species possibility through robust mating behavior and reproductive success studies.

Here, we discover *N. femorata* living, feeding, fighting and mating on thistle for the first time to our knowledge. *N. femorata* populations living on thistle and cactus plants have relatively similar body and weapon sizes as well as weapon composition. However, we found a difference in beak length between these two populations. This novel observation presents an excellent opportunity to see how a shift in host plant diet affects reproductive behaviors and survivorship which may contribute to our understanding of the evolution between insects and plants and the potential for speciation in this phytophagous species.

## AUTHOR CONTRIBUTIONS

**Lauren A. Cirino:** Formal analysis (lead); investigation (equal); validation (equal); visualization (lead); writing – original draft (lead). **Isaac McEvoy:** Data curation (lead); investigation (equal); methodology (equal); validation (equal). **Logan C. Smith:** Data curation (lead); investigation (equal); methodology (equal); validation (equal). **Zachary Emberts:** Conceptualization (lead); data curation (supporting); formal analysis (supporting); funding acquisition (lead); investigation (equal); methodology (equal); project administration (lead); resources (lead); supervision (lead); validation (equal); visualization (supporting); writing – original draft (supporting).

## FUNDING INFORMATION

Oklahoma State University funded this research.

## CONFLICT OF INTEREST STATEMENT

None.

## Supporting information


Data S1.

## Data Availability

Our data files and code used for analyses are included as electronic files in the Data S1.
